# Violencia obstétrica capacitista hacia mujeres con discapacidad: Una revisión integradora de la literatura

**DOI:** 10.18294/sc.2023.4676

**Published:** 2023-12-20

**Authors:** Pía Rodríguez-Garrido

**Affiliations:** 1 Doctora en Enfermería y Salud. Integrante del Núcleo Milenio Discapacidad y Ciudadanía, Chile; Grupo de Estudios Mujer, Salud y Ética, Universidad de Barcelona, España; Laboratório de Estudos Sociais sobre o Nascimento, Instituto Universitario de Lisboa, Portugal. Investigadora Postdoctoral, Instituto de Ciencias de la Salud, Universidad de O’Higgins, Rancagua, Chile. pia.rodriguez@uoh.cl Universidad de Chile Universidad de Chile Chile pia.rodriguez@uoh.cl; Núcleo Milenio Discapacidad y Ciudadanía Chile; Grupo de Estudios Mujer, Salud y Ética Universidad de Barcelona España; Laboratório de Estudos Sociais sobre o Nascimento Instituto Universitario de Lisboa Portugal; Instituto de Ciencias de la Salud Universidad de O’Higgins Rancagua Chile

**Keywords:** Capacitismo, Personas con Discapacidad, Violencia Obstétrica, Ableism, Disabled Persons, Obstetric ViolenceAbleism, Disabled Persons, Obstetric Violence

## Abstract

Las mujeres con discapacidad se ven enfrentadas a una mayor precariedad a lo largo de sus vidas. Una de las áreas más afectadas es su salud sexual y reproductiva. El objetivo de este estudio fue identificar y analizar la literatura sobre violencia obstétrica en mujeres con discapacidad. La búsqueda se realizó durante los meses de agosto a octubre de 2022 en cinco bases de datos: PubMed; Web of Science; Dialnet; SciELO y Scopus. Se recuperaron 194 artículos y luego de aplicar los criterios de selección se analizaron diez artículos. Del análisis temático, emergió la dimensión: “violencia obstétrica capacitista hacia mujeres con discapacidad”. Los hallazgos sugieren que las mujeres con discapacidad son invisibilizadas durante la atención obstétrica, lo que genera un cuidado inoportuno e intervenciones en cascada. Existe escasa literatura que aborde el fenómeno desde una perspectiva de derechos. Es urgente contar con equipos de salud aptos para atender a personas con discapacidad, así como también, problematizar el vínculo entre el personal e instituciones de salud y las mujeres con discapacidad en el marco de sus derechos sexuales y reproductivosa.

## Abstract (en)

Women with disabilities face increased precarity throughout their lives, with one of the most affected areas being their sexual and reproductive health. The aim of this study was to identify and analyze the scientific literature on obstetric violence against women with disabilities. The search was conducted from August to October 2022 in five databases: PubMed, Web of Science, Dialnet, SciELO, and Scopus. A total of 194 articles were retrieved, and after applying selection criteria, ten articles were analyzed. Through thematic analysis, the dimension “ableist obstetric violence towards women with disabilities” emerged. Findings suggest that women with disabilities are marginalized during obstetric care, resulting in untimely attention and cascading interventions. At present, there is scarce literature addressing this phenomenon from a rights-based perspective. Urgent measures are needed to train healthcare teams on the provision of care to people with disabilities. Additionally, it is crucial to critically examine the relationship between healthcare personnel and institutions to women with disabilities within the framework of their sexual and reproductive rights.

## INTRODUCCIÓN

Según datos de la Organización Mundial de la Salud (OMS), cerca del 15% de la población mundial manifiesta algún tipo de discapacidad, es decir, más de mil millones de personas. Esta cifra ha ido en aumento, ya que en 1970 el 10% de la población mundial presentaba algún tipo de discapacidad^1^^,^^2^. Respecto a ello, el Banco Mundial afirma que las personas en situación de discapacidad (término utilizado por el activismo para dar visibilidad al modelo social de la discapacidad, el cual plantea que son principalmente las barreras y actitudes del entorno las que impiden la plena participación de las personas) se ven enfrentadas a mayores adversidades en materia socioeconómica producto del difícil acceso a educación, a las malas condiciones de salud, y a las altas tasas de desempleo y pobreza^3^. 

Respecto a las mujeres con discapacidad la realidad es aún más adversa, ya que están expuestas a una mayor precarización en sus trayectorias de vida, sobre todo, en contextos geográficamente empobrecidos^3^. En estos escenarios las condiciones de habitabilidad y sobrevivencia se relacionan principalmente con la vulnerabilidad económica, cuestión que obstaculiza el acceso oportuno a atención sanitaria, a la entrega de cuidados y ayudas técnicas, al acceso a educación formal y a un empleo digno^4^.

El comité de expertos que supervisa la aplicabilidad de la Convención Internacional de los Derechos de las Personas con Discapacidad (CIDPD)^5^^,^^6^ señala, entre sus recomendaciones, especial preocupación por la autonomía, la discriminación, la violencia de género, y la vulneración de derechos sexuales y reproductivos que se ven enfrentadas las mujeres con discapacidad. A pesar de ello, las mujeres en situación de discapacidad -niñas, adolescentes, adultas y mayores- han visto sistemáticamente vulnerados sus derechos, particularmente, los derechos sexuales y reproductivos^7^. 

Dificultad en el acceso a educación sexual integral inclusiva, en la entrega de métodos anticonceptivos y de barrera, en el pleno disfrute y goce de su sexualidad, en el derecho de protección y reparación frente a actos de violencia obstétrica, y en el ejercicio libre de la maternidad, son solo algunos de los derechos que les han sido negados a las mujeres con discapacidad^8^^,^^9^.

Ante este panorama se ha generado un importante campo de investigaciones con el propósito de visibilizar y problematizar la vulneración de derechos sexuales y reproductivos de las mujeres con discapacidad. Estos se han enfocado en ámbitos como la sexualidad^7^, la anticoncepción^10^, la esterilización forzada^11^ y la maternidad^12^. Sin embargo, poco se ha investigado sobre las experiencias negativas asociadas a la atención de la salud sexual y reproductiva y las faltas de respeto tras ello, particularmente, es escaso el desarrollo epistémico en torno a la violencia obstétrica en mujeres con discapacidad^13^.

Este artículo es parte de los objetivos del proyecto de investigación “Mujeres en situación de discapacidad y sus procesos de maternidad (gestación, parto y postparto): experiencias en contextos rurales de Chile” (Fondecyt No. 3230576).

### Aproximaciones conceptuales y epistémicas de la violencia obstétrica

Dolores Ruiz-Berdun alude a una publicación del siglo XIX a cargo del obstetra inglés James Blundell como una de las primeras aproximaciones a la violencia obstétrica. En ella, el obstetra señala “Desbordes, tremendas laceraciones inversiones del útero, como las que ahora están sobre la mesa frente a ti. Tales son los efectos de la violencia obstétrica, una violencia feroz y atroz”^14^. 

El abordaje conceptual, teórico y político en torno a la violencia obstétrica ha sido desarrollado principalmente desde América Latina, así como desde las ciencias sociales y humanas pero, sobre todo, desde el activismo; esto ha permitido analizar el carácter estructural y complejo que involucra su alcance^15^^,^^16^^,^^17^^,^^18^.

A su vez, se ha incorporado legislativamente en al menos tres países de la región: Argentina, en seis estados de México y Venezuela; siendo en este último el primer país en legislar sobre violencia obstétrica en la Ley Orgánica sobre el derecho de la Mujer a una vida libre de violencia en 2007, donde la define como “la apropiación del cuerpo y los procesos reproductivos de las mujeres por parte del personal de la salud”^19^.

Organizaciones y colectivos sociales en la materia han denunciado enfáticamente la necesidad de instaurar en el debate público y político los malos tratos y faltas de respeto perpetuados hacia las mujeres en las atenciones obstétricas^20^. La excesiva medicalización durante la gestación, parto y postparto no es un evento aislado, del mismo modo que no se puede comprender unidimensionalmente, ya que implica una serie de sucesos que afectan directamente la integridad física, psicológica y emocional de las mujeres y sus familias^21^. 

Serena Brigidi (antropóloga médica, especialista en violencia obstétrica) señala en una de sus conferencias la similitud entre un abuso sexual y un parto medicamente intervenido. La similitud se infiere al mirar las fotografías de mujeres acostadas, desnudas, con ambas piernas abiertas y un hombre -médico ginecobstetra- frente a ellas tocándole sus genitales^22^. A esta situación, entre otras, se exponen sistemáticamente las mujeres con discapacidad durante sus procesos de gestación, parto y posparto, en tanto existe una “cultura institucional” que promueve y avala esas prácticas médicas^21^^,^^23^. Según Foucault uno de los principales fenómenos del siglo XIX fue la “consideración de la vida por parte del poder; por decirlo de algún modo, un ejercicio del poder sobre el hombre en cuanto ser viviente, una especie de estatización de lo biológico”^24^. Es esa biopolítica que controla el cuerpo y la subjetividad de las mujeres en las atenciones obstétricas.

### Cuando las mujeres con discapacidad experimentan la violencia obstétrica

Los estudios feministas de la discapacidad (*feminist disability studies)* han enunciado los discursos disidentes de las mujeres con discapacidad que se encontraban desplazados por el movimiento de personas con discapacidad y por los movimientos feministas^25^^,^^26^^,^^27^. Negación fuertemente criticada por activistas feministas de la discapacidad: “nos ofende que se silencie nuestras voces, de manera que no se reconozca la opresión que padecemos, y definimos como injusticia la exclusión de las personas discapacitadas del núcleo de la sociedad”^27^. 

Es así como los estudios feministas de la discapacidad han materializado las preocupaciones históricas de las mujeres con discapacidad, poniendo en el centro a las expertas por experiencia y sus procesos de de-construcción identitaria y colectiva. Es en ese camino que los estudios feministas de la discapacidad y los estudios sobre violencia obstétrica se entrecruzan al compartir el cuestionamiento a las relaciones de poder y la supeditación ante las estructuras de dominación^28^. A pesar de ello, las experiencias en torno a los procesos de gestación, parto y posparto de las mujeres con discapacidad han permanecido invisibilizadas en los estudios sobre violencia obstétrica.

Lo señalado es fundamental para pensar la posición política y de derecho de las mujeres con discapacidad y sus procesos de gestación, parto y posparto, ya que la construcción subalterna que les rodea se evidencia en su omisión desde los colectivos reivindicativos de mujeres^29^, en la subvaloración de la maternidad en el contexto social^30^^,^^31^, y en la interpelación constante al ejercicio de la maternidad por encontrarse en situación de discapacidad^32^^,^^33^. 

Respecto a ello, la maternidad ha significado un importante desafío para los colectivos feministas de la discapacidad en materia de demandas y políticas reivindicativas -Círculo Emancipador de Mujeres y Niñas con Discapacidad de Chile (CIMUNIDIS), Movimiento Feminista por la Accesibilidad Universal (FEMACU), Observatorio de Derechos Sexuales y Reproductivos de las Personas con Discapacidad (ODISEX)- ya que, a pesar de lo estipulado en el Artículo 23 de la CIDPD, que establece el respeto a decidir libremente la cantidad de hijos, el periodo intergenésico y el acceso oportuno a la educación reproductiva y de planificación familiar^5^, no se han podido garantizar estos principios en medidas concretas. Es en ese tránsito de tensiones y desafíos que emerge la necesidad de identificar y analizar la literatura sobre violencia obstétrica en mujeres con discapacidad.

## METODOLOGÍA

Se realizó una revisión integradora de la literatura, cuyo diseño permite generar un riguroso y sistematizado proceso de análisis de la información, a la vez que otorga una mirada crítica y reflexiva de la literatura encontrada. En palabras de Silamani Guirao Goris:

Este tipo de revisión, también denominada revisión crítica, tiene como objetivo demostrar que el autor ha investigado ampliamente la literatura y evaluado críticamente su calidad. Va más allá de la mera descripción de los artículos identificados e incluye un grado de análisis e innovación conceptual.^34^

Se optó por este tipo de revisión, ya que propone un enfoque analítico, se basa en una serie de etapas y criterios que resguardan la permanente problematización de la bibliografía encontrada. En esta revisión adoptamos las cinco etapas de desarrollo propuestas por Whittemore & Knalf^35^.

### Identificación del problema

Las mujeres con discapacidad están expuestas a una mayor precarización en sus trayectorias de vida. Dificultad en el acceso a educación sexual integral inclusiva, en el derecho de protección y reparación frente a actos de violencia obstétrica, y en el ejercicio libre de la maternidad, son algunos de los obstáculos a los cuales se enfrentan. Aun así, el abordaje epistémico-metodológico de los estudios que incorporan la violencia obstétrica no es erróneo, sino más bien omite e invisibiliza la realidad de las mujeres con discapacidad. Por este motivo, resulta fundamental identificar y analizar la literatura científica sobre violencia obstétrica en mujeres con discapacidad.

### Búsqueda bibliográfica

La búsqueda se realizó durante los meses de agosto a octubre de 2022, en las siguientes bases de datos: PubMed; Web of Science (WoS); Dialnet; SciELO y Scopus. Las combinaciones booleanas utilizadas en la búsqueda fueron: “*Obstetric violence* AND *disability*”; “*Violence* AND *motherhood* AND *disability*”; “*Violence* AND *childbirth* AND *disability*” en inglés. Además se utilizaron las siguientes combinaciones en español “Violencia obstétrica AND discapacidad”; “Violencia AND maternidad AND discapacidad”; “Violencia AND nacimiento AND discapacidad” (Tabla 1).

**Tabla 1:** Cantidad de artículos científicos recuperados según combinaciones de términos y base de datos (N = 194).

Base de datos	Obstetric violence AND disability	Violence AND Motherhood AND disability	Violence AND childbirth AND disability	Violencia obstétrica AND discapacidad	Violencia AND maternidad AND discapacidad	Violencia AND nacimiento AND discapacidad
PubMed	54	7	24	0	0	0
Web of Science	11	13	15	0	0	0
Dialnet	0	1	0	2	13	8
Scielo	0	0	0	1	0	0
Scopus	15	14	16	0	0	0

Fuente: Elaboración propia.

### Evaluación de los datos

**Figura 1:**
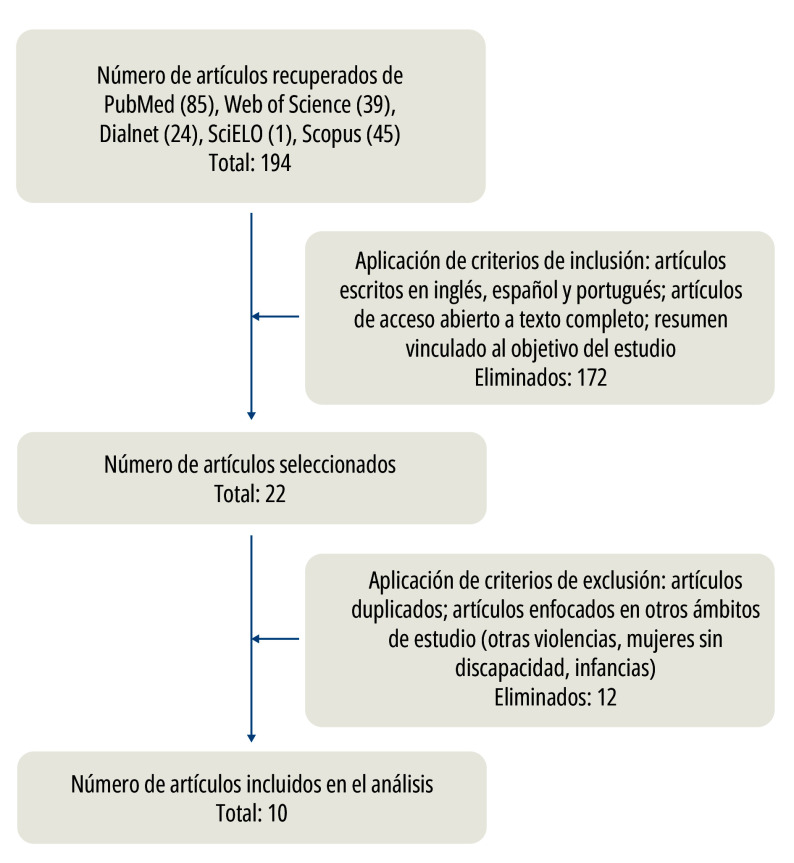
Flujograma de selección de artículos.

Los criterios de selección adoptados fueron: literatura científica que estuviera escrita en inglés, español y portugués, que tuviera relación con el objetivo de estudio y que fuera de acceso abierto, es decir, que no hubiera que pagar para su lectura y descarga (Figura 1). Por su parte, los criterios de exclusión fueron: artículos duplicados en bases de datos y que estuvieran enfocados en otros ámbitos de estudio, por ejemplo, en otros tipos de violencias, que no contemplaran a mujeres con discapacidad o bien que incorporaran a infancias en sus análisis (Figura 1).

Fuente: Elaboración propia.

### Análisis de los datos

El análisis se llevó a cabo en dos etapas: a) primero se realizó una lectura de títulos y resúmenes de los 194 documentos seleccionados, luego se realizó una segunda lectura en detalle que permitiera generar un análisis en profundidad del fenómeno de estudio; b) la segunda etapa consistió en analizar temáticamente^36^ los diez artículos seleccionados (Tabla 2) y agruparlos en una gran dimensión de análisis titulada “violencia obstétrica capacitista hacia mujeres con discapacidad”.

**Tabla 2:** Información de los artículos seleccionados para el análisis.

Año publicación	Autores	País del estudio	Título del estudio	Objetivo	Conclusiones
2021	James Gordon Rice; Helga Baldvins Bjargardóttir; Hanna Björg Sigurjónsdóttir	Islandia	Child protection, disability and obstetric violence: Three case studies from Iceland	Identificar, a través de un marco longitudinal y comparativo, por qué estas dificultades persisten a pesar de un entorno cambiante en materia de derechos de las personas con discapacidad.	El concepto de violencia obstétrica es un marco útil para la crítica y el trabajo de incidencia en esta área.
2022	Aregahegn Wudneh, Aneleay Cherinet, Mesfin Abebe, Yesuneh Bayisa, Nebiyu Mengistu and Wondwosen Molla.	Etiopía	Obstetric violence and disability overlaps: obstetric violence during childbirth among women with disabilities: a qualitative study.	Explorar la experiencia de las mujeres con discapacidad frente a la violencia obstétrica durante el parto en la zona de Godio, en el sur de Etiopía.	La calidad del servicio es deplorable, y las denuncias de violencia obstétrica entre este grupo vulnerable de mujeres les imponen una doble carga. Es necesario mejorar la atención de maternidad de las mujeres con discapacidad mediante la implementación de servicios especiales integrales, culturalmente sensibles y capacitar a los proveedores de atención médica, asegurando una atención obstétrica satisfactoria, equitativa y de calidad.
2019	Hridaya R. Devkota, Maria Kett and nora Groce	Reino Unido	Societal attitude and behaviours towards women with disabilities in rural Nepal: pregnancy, childbirth, and motherhood.	Examinar las actitudes y los comportamientos de la sociedad rural nepalesa hacia las mujeres con discapacidad, su embarazo, parto y maternidad.	Las mujeres con discapacidad se enfrentan a importantes desafíos por parte de la familia y la sociedad en todas las esferas de su vida reproductiva, incluidos el embarazo, el parto y la maternidad. Es necesario que la política social aumente la conciencia pública y mejore las actividades de promoción para mitigar los conceptos erróneos sobre la discapacidad y promover los derechos sexuales y reproductivos de las mujeres con discapacidad.
2012	Dana Sumilo, Jennifer J Kurinczuk, Maggie E Redshaw and Ron Gray.	Reino Unido	Prevalence and impact of disability in women who had recently given birth in the UK.	Proporcionar una visión general de la prevalencia de la discapacidad en las mujeres que dan a luz en el Reino Unido, medida por la presencia de una enfermedad limitante de larga duración (LLI)	La discapacidad se asocia con desigualdades sociales y económicas y peores resultados relacionados con el embarazo y los hijos. Además del apoyo específico para cada afección durante y después del embarazo, ya que las mujeres con discapacidad pueden necesitar ayuda adicional de los profesionales de la salud para dejar de fumar, continuar amamantando y reducir la violencia de pareja.
2012	Ruchira Naved; Lauren Blum; Sadia Chowdhury; Rasheda Khan; Sayeda Bilkis; Marge K	Bangladesh	Violence against Women with Chronic Maternal Disabilities in Rural Bangladesh	Explorar la violencia contra las mujeres madres con discapacidades crónicas en las zonas rurales de Bangladesh.	Es necesario elaborar iniciativas para abordar las percepciones erróneas sobre las causas de esas discapacidades y, a largo plazo, crear oportunidades económicas para reducir la dependencia de las mujeres del matrimonio y los hombres y transformar la sociedad para superar las rígidas normas de género.
2019	Elaine Xie; Meg Gemmill	Canadá	Exploring the prenatal experience of women with intellectual and development disabilities. In a southeastern Ontario family health team	Identificar los desafíos psicosociales a los que se enfrentan las mujeres embarazadas con discapacidades intelectuales y del desarrollo (IDD) utilizando datos retrospectivos de historias clínicas electrónicas recopilados de forma rutinaria.	Los hallazgos de este estudio respaldan investigaciones anteriores de que las mujeres embarazadas con IDD son una población vulnerable, con un mayor riesgo de resultados adversos para la salud. Se necesitan pautas de atención específicas para los proveedores de atención médica, así como recursos adicionales y apoyo social.
2016	María del Pilar Gomiz Pascual	España	La sexualidad y la maternidad como factores adicionales de discriminación (y violencia) en las mujeres con discapacidad.	Analizar la violencia que existe contra las mujeres con discapacidad en sus diferentes vertientes, con el objetivo de visibilizar a un grupo social especialmente vulnerable.	Son recurrentes episodios violentos que van desde la negación de cuidados, la humillación o la estigmatización por la discapacidad.
2022	Andrea Yupanqui-Concha; Melissa Hichins Arismendi; Daniela Mandiola Godoy.	Chile	“Yo fui violentada adentro, estando en un lugar que me tenían que cuidar”: Experiencias de opresión y violencias en contextos de salud hacia mujeres con discapacidad y abordajes desde la terapia ocupacional feminista	Caracterizar prácticas de violencia hacia mujeres con discapacidad en contextos de salud y caracterizar experiencias de reivindicación de derechos humanos de este colectivo de mujeres en Chile.	Las prácticas de violencia en contextos de salud hacia mujeres con discapacidad en Chile es una situación visualizada como manifestaciones de dominación y opresión contra ellas, que perpetúan su exclusión social y desigualdades en salud.
2022	Dinke Aga Hirpa	Etiopía	Sexual violence and motherhood among women with disabilities in Ambo Town, Ethiopia	Explorar la violencia sexual experimentada por mujeres con discapacidades físicas.	Es fundamental tomar medidas para crear conciencia y diseñar políticas que garanticen que se respeten los derechos de las mujeres con discapacidad a la maternidad en Etiopía y se mejore la calidad de sus vidas.
2021	Betty Kwagla and Stephen Ojiambo Wandera	Uganda	The determinants of early childbearing by disability status in Uganda: an analysis of demographic and health survey data.	Evaluar las determinantes de la maternidad precoz entre las mujeres según el estado de discapacidad	Las medidas deben dirigirse a las mujeres con discapacidad, independientemente de sus actitudes hacia la violencia, de sus conocimientos sobre la fecundidad y de su región.

Fuente: Elaboración propia.

## RESULTADOS

### Dimensión de análisis: “Violencia obstétrica capacitista hacia mujeres con discapacidad”

El capacitismo “es una categoría que define cómo las personas con discapacidad son generalmente tratadas como incapaces (incapaces de producir, trabajar, aprender, amar, cuidar, sentir deseos, tener relaciones sexuales, etc.)”^37^. Bajo esta comprensión, la violencia obstétrica capacitista radica en un conjunto de prácticas abusivas y negligentes por parte del equipo de salud hacia las mujeres con discapacidad, producto de los estereotipos y representaciones negativas en torno a la incapacidad de autonomía, respecto a su salud sexual y reproductiva. Alertar este tipo de violencia otorga lineamientos y problematizaciones que nos ayudan a identificar y reconocer las trayectorias vitales de vulneración y precariedades que experimentan las mujeres con discapacidad.

En ese sentido, la institucionalidad es un espacio de tensión constante para las mujeres con discapacidad. La ausencia de discernimiento, la asexualidad y la infantilización son algunas de las premisas arraigadas en el discurso médico que vulneran sus derechos sexuales y reproductivos e impiden el ejercicio autónomo de sus maternidades^7^^,^^12^. 

Wudneh *et al*.^13^ señalan que la violencia obstétrica es un grave problema estructural, situándolo como un tipo de violencia sexual hacia las mujeres. Añaden que se ejerce principalmente en mujeres en condiciones vulnerables: racializadas, migrantes, empobrecidas, jóvenes y con discapacidad. Una de las manifestaciones explícitas en este colectivo es la realización de cesáreas para luego esterilizarlas sin su conocimiento; no obstante, también experimentan: abuso físico, verbal, estigma y discriminación, negligencias y abandono, violación a su privacidad y autonomía. La sensación de ser tratadas como “no humanas” o “animales”, o la “invisibilización de sus cuerpos y decisiones” son algunas de las declaraciones que señalan los autores en sus hallazgos.

Respecto a los procesos de maternidad, específicamente en el periodo posparto, Rice, Bjargardóttir y Sigurjónsdottir^38^ señalan que, en el caso de las madres con discapacidad, existe una mayor presión por otorgar un cuidado de calidad hacia sus hijos en comparación con las madres sin discapacidad. Más aún, los actos discriminatorios y el estigma asociado a la discapacidad se agudizan en mujeres que tienen una discapacidad intelectual, ya que pone en constante tensión la presencia de competencias idóneas para ejercer el rol de buena madre. Otra manifestación explícita de violencia obstétrica se evidencia cuando a las madres se les quita la custodia de sus hijos por el hecho de tener discapacidad. Respecto a ello, constantemente se les está interpelando sobre la calidad de los cuidados y si son “capaces” o no de ejercer el rol de madres. Enfatizan en que los errores que puede cometer cualquier madre en el cuidado se magnifican cuando las mujeres tienen discapacidad intelectual, sobre todo, si son madres por primera vez. Según los autores, esta es la excusa perfecta para involucrar a los servicios sociales de cuidados de menores y poner en tensión la custodia de sus hijos.

Devkota, Kett y Groce^39^ describen la permanente devaluación que experimentan las mujeres con discapacidad en sus procesos de gestación, parto y crianza, limitando sus derechos sexuales y reproductivos. Particularmente, los autores señalan una serie de dificultades asociadas a la capacidad de concretar un matrimonio, concebir, tener un parto y otorgar cuidados a sus hijos en comparación con las mujeres sin discapacidad. Así mismo, señalan que la sociedad patriarcal en la cual están insertas no colabora en los procesos de inclusión de las mujeres con discapacidad. Específicamente, en Nepal, describen que el acceso a la educación, el matrimonio, el empleo y la participación política de las mujeres con discapacidad se ve radicalmente obstaculizada. Sus familiares, vecinos y cercanos, constantemente devalúan su posición como sujetas de derecho. 

Respecto a la preparación del equipo de salud que acompaña la gestación y parto de las mujeres con discapacidad, Šumilo *et al*.^40^ señalan la importancia de que debe ser un equipo preparado y respetuoso. Esto podría ser la norma, en tanto característica inherente al personal de salud que otorga atenciones obstétricas a mujeres con discapacidad pero, sin embargo, no lo es.

Respecto a ello, la investigación de Naved *et al*.^41^ analizó las diversas manifestaciones de violencias a las cuales se ven enfrentadas las mujeres con discapacidad en Bangladesh quienes, producto de diversas intervenciones médicas realizadas durante el proceso de parto, quedaron con una discapacidad crónica como secuela (por ejemplo, prolapso uterino, pérdida involuntaria de orina, o una fístula vesicovaginal). Según los autores, esta situación se da con mayor frecuencia en países empobrecidos, donde el acceso a una salud de calidad es un privilegio más que un derecho. Sobre esto, Kwagala y Wandera^42^, en su estudio realizado en Uganda, describen que la maternidad de las mujeres con discapacidad se ve obstaculizada, ya que se da en condiciones de baja educación, abuso sexual, matrimonios arreglados y limitado acceso a métodos anticonceptivos.

Por su parte, respecto a las mujeres con discapacidad intelectual, una de las manifestaciones de violencia obstétrica más frecuente de observar se relaciona con la esterilización forzada. Xie y Gemmill^43^ señalan que, en Canadá, EEUU y algunos países de Europa, la esterilización forzada era institucionalmente legal y aceptada a mediados del siglo XX. Dentro de las justificaciones obstétricas para realizarla se encuentran la preeclampsia, el parto prematuro y la cesárea de urgencia; sin embargo, los autores vislumbran otras causas asociadas que se vinculan con la capacidad de ejercer cuidados a los hijos y su correcta custodia, más que a causas nétamente médicas o biológicas.

Otra compleja situación que evidencia la esterilización forzada tiene relación con historias de abuso y violencia sexual hacia las mujeres con discapacidad, principalmente intelectual^44^^,^^45^^,^^46^^,^^47^^,^^48^. En algunos casos son historias que datan de la infancia o adolescencia y son perpetuados por amigos o miembros de la familia. Xie y Gemmill^43^ añaden que, de las diez participantes de su estudio, siete presentaron historial médico de abuso sexual, físico o verbal. Respecto a esto, Hirpa^49^ argumenta que la mayoría de las mujeres con discapacidad física, participantes de su estudio, fueron madres producto de violaciones o de actos sexuales sin su consentimiento. Añadiendo que, a la dificultad de entablar relaciones amorosas y consentidas, se incluyen las barreras sociales asociadas a sus procesos de maternidad en tanto traumas que emergen como consecuencia de abusos y agresiones sexuales. 

Respecto a la caracterización de las diversas manifestaciones de violencia a las cuales se ven expuestas las mujeres con discapacidad, Andrea Yupanqui-Concha, Melissa Hichis Arismendi y Daniela Mandiola Godoy^50^ identifican cinco dimensiones en las cuales se alojan estas prácticas: violencia física, violencia psicológica y/o emocional, violencia sexual, violencia obstétrica y violencia simbólica-institucional. Respecto a la violencia obstétrica, las autoras diferencian los procedimientos forzados irreversibles ejercidos por un profesional de la salud, como el aborto y la esterilización forzada, y las intervenciones forzadas de carácter reversible, como la anticoncepción transitoria forzada y las amenazas y discriminación vinculadas a la maternidad. En la misma línea, Gomíz Pascual^51^ añade que además de la esterilización forzada, se incluyen otras prácticas lesivas en el ejercicio de la maternidad, como los abortos coercitivos. Así mismo, señala que la dependencia económica y la violencia ejercida por parte de la pareja íntima es una diada frecuente de encontrar, vinculado a la dificultad de las mujeres con discapacidad para acceder a trabajos y remuneración digna. 

## DISCUSIÓN

Los hallazgos de este estudio aluden a la violencia obstétrica capacitista como prácticas abusivas y negligentes provocadas por el equipo médico hacia las mujeres con discapacidad en el marco de la atención obstétrica. En ese marco, según lo descrito con Pino-Morán^52^, la violencia institucional capacitista “establece patrones y mandatos normativos sobre los cuales se conducen formas de gobierno y subjetividad. A partir de esto se puede establecer qué cuerpos son legítimos y cuales son ilegítimos”^52^. Justamente es esa legitimidad la que se tensiona cuando se realizan prácticas interventivas en los cuerpos y subjetividades de las mujeres con discapacidad sin su consentimiento.

A este respecto, la violencia obstétrica, como expresión de violencia de género durante los procesos de gestación, parto y posparto, comprende la realización de una serie de intervenciones desaconsejadas por organismos internacionales^53^. Dentro de las prácticas más recurrentes se encuentran la aplicación de oxitocina sintética (hormona exógena que provoca contracciones uterinas), la maniobra de Kristeller (presión del fondo uterino para apresurar la expulsión del feto), la episiotomía (corte en el sector del periné para ampliar el canal vaginal) o la cesárea de rutina. No obstante, cuando estas prácticas se llevan a cabo en las mujeres con discapacidad, curiosamente no son catalogadas como actos de violencia obstétrica, sino más bien como necesarias para su bienestar y el de sus hijos e hijas. Es aquí donde la legitimidad y la autonomía de sus cuerpos se ve mermada.

En ese sentido, y de acuerdo a los hallazgos, la institucionalidad cumple un rol particular en la promoción de dinámicas de poder y violencia que se dan en su interior. Esto, según algunos autores^21^^,^^23^ responde a la cultura institucional propia del modelo médico presente en las instituciones de salud, ya que una de sus principales características es que cumple “funciones de normatización, de control y de legitimación”^54^. Lo señalado es especialmente alarmante en las mujeres con discapacidad, ya que al comprenderse imperfectas e inacabadas desde el modelo médico, deben ser necesariamente intervenidas y reparadas^27^. De ahí que Wudneh *et al*.^13^^)^ señalen la realización de cesáreas de rutina con el propósito de esterilizar quirúrgicamente a las mujeres con discapacidad sin mayor información o consentimiento. 

La esterilización forzada es una intervención ampliamente realizada en mujeres y niñas con discapacidad^43^^,^^50^. Según la Relatora Especial de las Naciones Unidas sobre los derechos de las personas con discapacidad, la esterilización es una intervención muy frecuente, incluso hasta tres veces mayor que en la población general, así mismo, afirma que es una modalidad de tortura, y que constituye una violación a los derechos humanos^55^. 

Esta manifestación de violencia obstétrica no solo las anula en su calidad como sujetas de derecho, sino que además provoca lo que Massó enuncia como “injusticia epistémica en la atención obstétrica”^56^, esa injusticia testimonial y hermenéutica tras el exceso o carencia de información, prácticas e intervenciones, donde el capacitismo es la retórica que justifica el maltrato hacia las mujeres con discapacidad. 

Por su parte, Rice, Bjargardóttir y Sigurjónsdottir^38^^)^ describen la constante presión que sufren las mujeres con discapacidad por demostrar sus competencias como buenas madres; construcción cultural ampliamente desarrollada por los estudios de maternidad y maternaje (*mothering and motherhood studies*)^31^. De acuerdo a estos, el rol de la buena madre se instaló a principios del siglo XVIII con la promoción de la puericultura, de la lactancia materna y de los cuidados hacia el recién nacido en respuesta a una motivación higienista y eugenésica de la época, no obstante, esta promoción pondría el foco principalmente en mujeres re-productivas, útiles al capital^31^. De este modo, las mujeres con discapacidad quedarían fuera de este estereotipo, al no cumplir con la representación social para ejercer el rol de la buena madre, en tanto se las vincula a la imposibilidad de gestar y parir, a cuidados negligentes, a la infantilización de su maternidad, a la heredabilidad de la discapacidad y a la incapacidad productiva. Lo descrito, además de ser un recurso útil para las instituciones médicas, es la excusa perfecta de los servicios sociales para tensionar la custodia de sus hijos y la posibilidad de perder su tuición^33^.

Otra manifestación de violencia obstétrica, según los hallazgos, tiene relación con la permanente devaluación que experimentan las mujeres con discapacidad en sus procesos de gestación, parto y crianza limitando sus derechos sexuales y reproductivos. La filósofa Sara Cohen denomina *gaslighting* en la atención obstétrica^57^^)^ a la omisión de los cuerpos y decisiones de las mujeres durante el proceso de parto. Se manifiesta por parte del personal de salud y, en ocasiones, por parte de los propios familiares de las mujeres gestantes, al negar las experiencias no gratas durante el nacimiento y omitir las emociones negativas que emanan de aquello, minorizando la situación o haciéndoles creer que exageran en su interpretación. Esto se agudiza en las mujeres con discapacidad, ya que se añaden otras características, como el estigma de la discapacidad, lo cual potencia la anulación de sus vivencias^13^^,^^44^^,^^45^^,^^46^^,^^47^^,^^48^.

Por último, de acuerdo a Naved *et al*.^41^, Kwagala y Wandera^42^, el bajo nivel educacional y socioeconómico, así como la ubicación geopolítica, inciden directamente en el sistema de salud y la calidad de la atención, según los autores, es más probable que una mujer con discapacidad sufra violencia obstétrica en un sector geopolíticamente empobrecido que en otro contexto geográfico. Panorama similar identifica el Banco Mundial al señalar que las mujeres con discapacidad se ven enfrentadas a mayores adversidades producto del difícil acceso a educación, a las malas condiciones de salud, y a las altas tasas de desempleo y pobreza, sobre todo en escenarios geográficamente empobrecidos^3^. 

No obstante, a la adversidad del contexto, Šumilo *et al*.^40^ señalan que el equipo de salud que atiende a personas con discapacidad, específicamente, a mujeres con discapacidad, debe ser personal preparado y respetuoso. En ese sentido, el Servicio Nacional de la Discapacidad de Chile señala la importancia de los ajustes razonables y la accesibilidad en la atención sanitaria^58^, a su vez, se promueve que sea una atención basada en un enfoque de género, de derechos e inclusiva, al tratarse de aspectos complejos como la salud sexual y reproductiva^59^.

### Limitaciones del estudio

Se puede inferir que ampliar las combinaciones o incluir sinónimos de violencia obstétrica, como falta de respeto o maltrato durante la atención de salud, pudiese haber ampliado la selección de la literatura en la materia. Por otra parte, haber seleccionado solo artículos de acceso abierto puede haber dejado fuera información relevante, presente en artículos de pago. 

## REFLEXIONES FINALES

Este estudio permitió identificar y analizar la literatura científica sobre violencia obstétrica en mujeres con discapacidad. A pesar de la escases de estudios que reflejen estas experiencias, se obtuvieron interesantes hallazgos que permitieron complejizar el fenómeno desde distintas aristas. En ese sentido, la violencia obstétrica es una manifestación de violencia de género, por ende, su realización por parte del equipo de salud no tan sólo afecta la dimensión reproductiva, sino también, el desarrollo, independencia y autonomía de las mujeres con discapacidad en tanto sujetas de derecho. 

La violencia obstétrica en las mujeres con discapacidad contempla tanto la obstaculización del ejercicio de la maternidad como su negación. Así, los métodos anticonceptivos y de barrera, la esterilización forzada y los abortos coercitivos, son intentos eugenésicos de evitar explícitamente la reproducción de las mujeres con discapacidad. No obstante, cuando son madres, se les reduce, infantiliza y coarta de su rol, incluso en algunos casos se pone en duda el cuidado y custodia de sus hijos.

A pesar de que la literatura analizada señala una indudable problemática tras los malos tratos en la atención obstétrica hacia mujeres con discapacidad -sobre todo intelectual-, es una realidad poco abordada y problematizada, incluso por los Estados parte que han ratificado la Convención de los Derechos de las Personas con Discapacidad. Convención que, cabe destacar, busca resguardar y promover los derechos sexuales y reproductivos de las personas con discapacidad. 

Los desafíos en la materia son evidentes. Es fundamental fortalecer el corpus teórico y analítico del fenómeno, sobre todo desde las propias protagonistas. A su vez, es imperante concientizar a los equipos de salud respecto a la atención oportuna de las mujeres con discapacidad, promoviendo la incorporación de ajustes razonables, ayudas técnicas y enfoque de derechos. Del mismo modo, como en otros contextos que afectan a las mujeres con discapacidad y su posición como ciudadanas, es fundamental considerarlas en los procesos de toma de decisiones y en el diseño y desarrollo de políticas públicas como expertas por experiencia. Por último, es prioritario que la institucionalidad médica, jurídica y educacional, que velan por la erradicación de la violencia de género y la violencia obstétrica, tomen consciencia respecto a los derechos sexuales y reproductivos de las mujeres con discapacidad, ya que el buen trato y el respeto por la autonomía y dignidad no debe ser un privilegio de algunas, sino un derecho de todas.
